# Genome-wide screening of DNA methylation associated with lymph node metastasis in esophageal squamous cell carcinoma

**DOI:** 10.18632/oncotarget.17147

**Published:** 2017-04-17

**Authors:** Hiroaki Nagata, Ken-Ichi Kozaki, Tomoki Muramatsu, Hidekazu Hiramoto, Kousuke Tanimoto, Naoto Fujiwara, Seiya Imoto, Daisuke Ichikawa, Eigo Otsuji, Satoru Miyano, Tatsuyuki Kawano, Johji Inazawa

**Affiliations:** ^1^ Department of Molecular Cytogenetics, Graduate School, Tokyo Medical and Dental University, Tokyo, Japan; ^2^ Hard Tissue Genome Research Center, Graduate School, Tokyo Medical and Dental University, Tokyo, Japan; ^3^ Bioresource Research Center, Graduate School, Tokyo Medical and Dental University, Tokyo, Japan; ^4^ Genome Laboratory, Graduate School of Medicine, Graduate School, Tokyo Medical and Dental University, Tokyo, Japan; ^5^ Department of Esophageal and General Surgery, Graduate School, Tokyo Medical and Dental University, Tokyo, Japan; ^6^ Department of Digestive Surgery, Graduate School of Medical Science, Kyoto Prefectural University of Medicine, Kamigyo-ku, Kyoto, Japan; ^7^ Human Genome Center, Institute of Medical Science, University of Tokyo, Tokyo, Japan; ^8^ Department of Dental Pharmacology, Graduate School of Medicine, Dentistry and Pharmaceutical Sciences, Okayama University, Kita-ku, Okayama, Japan

**Keywords:** esophageal squamous cell carcinoma, DNA methylation, predictive biomarker, lymph node metastasis, pyrosequencing analysis

## Abstract

Lymph node metastasis (LNM) of esophageal squamous cell carcinoma (ESCC) is well-known to be an early event associated with poor prognosis in patients with ESCC. Recently, tumor-specific aberrant DNA methylation of CpG islands around the promoter regions of tumor-related genes has been investigated as a possible biomarker for use in early diagnosis and prediction of prognosis. However, there are few DNA methylation markers able to predict the presence of LNM in ESCC. To identify DNA methylation markers associated with LNM of ESCC, we performed a genome-wide screening of DNA methylation status in a discovery cohort of 67 primary ESCC tissues and their paired normal esophageal tissues using the Illumina Infinium HumanMethylation450 BeadChip. In this screening, we focused on differentially methylated regions (DMRs) that were associated with LNM of ESCC, as prime candidates for DNA methylation markers. We extracted three genes, *HOXB2*, *SLC15A3*, and *SEPT9*, as candidates predicting LNM of ESCC, using pyrosequencing and several statistical analyses in the discovery cohort. We confirmed that *HOXB2* and *SEPT9* were highly methylated in LNM-positive tumors in 59 ESCC validation samples. These results suggested that *HOXB2* and *SEPT9* may be useful epigenetic biomarkers for the prediction of the presence of LNM in ESCC.

## INTRODUCTION

Esophageal squamous cell carcinoma (ESCC) is one of the most lethal cancers in spite of recent advancements in its diagnosis and therapeutics [1, 2]. One of the reasons for its poor prognosis is due to the development of lymph node metastasis (LNM) in the early phases [3]. Thus, early diagnosis and treatment are essential for ESCC patients. Predicting the presence of LNM before treatment is important for deciding appropriate therapy. Although endoscopic submucosal resection (ESD) is useful for minimally invasive treatment of ESCC, adaptation of the method is very limited. Indication of ESD requires early stage ESCC, in which the rate of LNM is low. If ESD is not indicated, esophagectomy with lymph node dissection is chosen as the next alternative for radical treatment. However, most patients are diagnosed at late-stage, and are often accompanied with extensive lymph node metastases. The degree of lymph node metastasis has been regarded as the most important prognostic factor in late stage disease [4, 5].

The presence and degree of lymph node metastasis have important clinical implications for the selection of proper treatment in patients with esophageal cancer. However, current imaging modalities, such as multi-detector-row computed tomography (MDCT), endoscopic ultrasonography (EUS) and positron emission tomography (PET), have limited diagnostic capabilities for precise detection of regional lymph node metastasis. The diagnostic sensitivity of MDCT for LNM, which is frequently used for ESCC staging, is said to be approximately 68%. If the presence of LNM can be predicted more precisely during the diagnostic phase prior to treatment, unnecessary invasive treatment can be avoided. However, despite numerous studies, no clinically useful LNM markers for ESCC have emerged. Thus, a powerful and less invasive LNM marker with high predictive power is required [6–8].

DNA methylation is one of the fundamental epigenetic processes. An epigenetic change is defined as a stable alteration in gene expression with no underlying modifications in the genetic sequence. Several epigenetic mechanisms regulate gene expression, including DNA methylation, histone modification, and non-coding RNA [9]. DNA methylation is one of the most widely and intensively studied processes in cancer epigenetics [10–12]. DNA hypermethylation involves the binding of methyl groups to CpG dinucleotides in the promotor region of a gene, controlling its expression. Aberrant DNA methylation of CpG islands in the promotor region of tumor suppressor genes has been widely reported in several types of cancer [13, 14]. Similarly, there have also been many studies on aberrant DNA methylation in ESCC [15]. Furthermore, some differentially methylated regions (DMR) associated with LNM in ESCC have previously been reported, such as *UCHL1* [16, 17] and *RARβ* [18, 19].

These findings prompted us to investigate LNM-associated DMR, which may be useful for the selection of appropriate treatment in ESCC patients [20]. We performed genome-wide screening of DMR associated with LNM in ESCC patients, and extracted 10 candidate genes using methylation array data of 67 ESCC samples in a discovery cohort. Subsequently, *Homeobox B2* (*HOXB2*), *Solute carrier family 15 member 3* (*SLC15A3*), and *Septin 9* (*SEPT9*) were narrowed down as final candidate genes for predicting LNM after several statistical analyses of the discovery cohort. The three candidate genes were validated as LNM predictive markers using 59 ESCC samples from another cohort. Finally, we identified *HOXB2* and *SEPT9* as LNM predictive markers by observing their methylation status. Thus, evaluation of *HOXB2* and *SEPT9* methylation status may facilitate earlier diagnosis of LNM in patients with ESCC.

## RESULTS

### Genome-wide screening of differentially methylated regions associated with lymph node metastasis in ESCC

**Figure 1:**
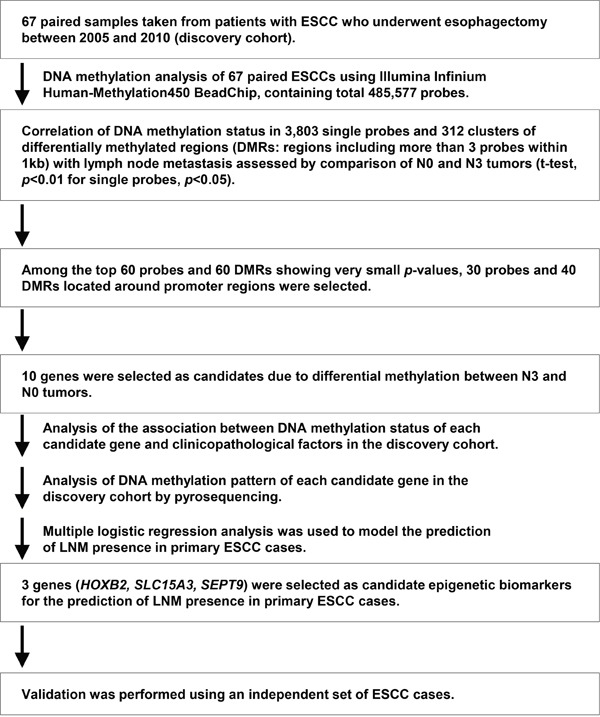
Schematic strategy for the identification of useful epigenetic biomarkers for the prediction of the presence of LNM in primary ESCC cases

To identify LNM-associated epigenetic biomarkers, we utilized the Illumina Infinium Human Methylation450 BeadChip array (Figure 1). Methylation information of the genome was obtained for a total of 485,577 CpG sites in 67 tumor and non-tumor paired ESCC frozen samples (Supplementary Table 1). The data were qualified by Genome studio software, and the output was saved as tab-separated files. The degree of cytosine methylation was scored by the beta value, which is the intensity ratio of methylated and unmethylated probes for each CpG site, ranging from 0 (unmethylated) to 1 (methylated). Sixty-seven samples were classified by N stage, and methylation profiles of representative genes are shown in Supplementary Figure 1A. Differences between beta values of tumor and normal tissue pairs, defined as the delta beta value and ranging from -1 to 1, were investigated to identify hyper- and hypo-methylation induced by carcinogenesis (Supplementary Figure 1B). Probes showing significant differences in delta beta values between N0 and N3 patients were identified as possible candidate predictors of LNM. Two approaches were utilized for extraction of candidate probes: identification of (i) single probes showing methylation status differences and (ii) probe clusters (groups of probes located within 1,000 bp of each other) showing methylation status differences. In the former approach, Student's *t*-test was performed and average delta beta values of all single probes were compared between the N0 and N3 groups, identifying 3,803 significantly methylated probes in the N3 group compared with the N0 group (p < 0.01). We have confirmed the normality of the data by Shapiro-Wilk test before Student's *t*-test. Based on *p*-value, six genes were selected as candidates: *NEFL*, *SLC15A3*, *OBSL1*, *PLEC1*, *SEPT9*, and *HOXD9*. The methylation profile heat map of each candidate gene is shown in Supplementary Figure 1A and 1B. For the latter approach assessing clustered methylation status, Student's *t*-test was performed in the same manner, and average delta beta values of all clusters were compared between the N0 and N3 groups. Three hundred and twelve probes with significant differences (*p* < 0.05) in methylation status were extracted and visualized as a heat map. Since we repeated hypothesis testing, some correction like Bonferroni's correction is necessary. However, we will create multivariate model for predicting LNM status and we want to minimize the false negatives. This is the reason why we used this relaxed significant level. Consequently, four genes, *HOXB2*, *PAX6*, *MIR124-2*, and *KDM2B*, were extracted (Supplementary Figure 1A and 1B). Thus, a total of 10 candidate genes were extracted as possible predictive markers of LNM by methylation array analysis (Supplementary Table 2).

### Extraction of candidate genes for prediction of LNM by methylome analysis of the discovery cohort

**Figure 2:**
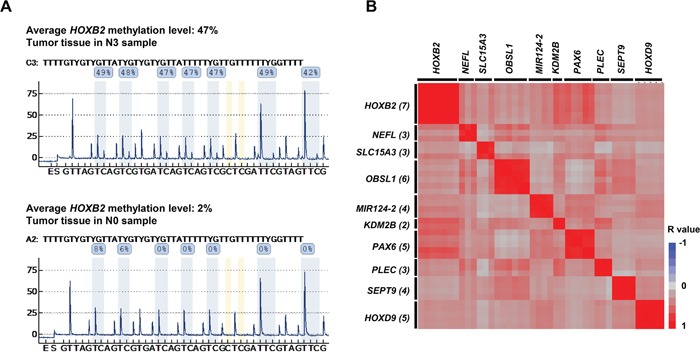
DNA methylation analysis by pyrosequencing **(A)** Pyrosequencing was performed to measure the methylation level of candidate genes to validate the Illumina HumanMethylation450 assay results. Candidate CpGs in *HOXB2* are shown. Average methylation was higher in N3 tumor tissue samples (upper: 47%) than in N0 tumor tissue samples (lower: 2%). **(B)** Correlation diagram of pyrosequencing data of each CpG site of the candidate genes. Matrix shows the correlation coefficient (r: -1 [blue] to 1 [red]) among all CpG sites within the sequencing areas of the pyrosequencing analysis. Each candidate gene contained multiple CpG sites. Rows and columns represent each CpG site of each candidate gene. The numbers in parentheses after gene name represent the number of CpG site which were within the sequence analyzed.

Pyrosequencing analysis was performed to validate the candidate genes extracted from the methylation array analysis. Pyrosequencing probes were designed using Pyromark Assay Design software to include all candidate probes. As an example, for *HOXB2*, three unique Illumina probes harboring target CpG sequences within the candidate gene were extracted by methylation array analysis (Supplementary Table 2), and forward and reverse primers were designed to cover all extracted array probes for PCR (Supplementary Table 3). Pyrosequencing allows assessment of CpG sequences in PCR products, and the methylation status of all CpG sites in the target regions of each candidate gene could be obtained. In the *HOXB2* gene, seven CpG sites were identified within the designed sequence range (Figure 2A). The methylation statuses of all candidate genes were measured in both the tumor and non-tumor samples by pyrosequencing analysis, and compared in each tumor (all N stages) and non-tumor pair to evaluate the usefulness of these candidate genes as diagnostic biomarkers. Figure 2A shows representative results for *HOXB2* in an N3 sample (upper), with hypermethylation, and an N0 sample (bottom). Subsequently, we evaluated the correlation among all probes in the extracted candidate genes, and observed that no probe showed correlation with any other probe (Figure 2B). Thus, the probes of each candidate gene may be useful as independent methylation markers.

**Figure 3:**
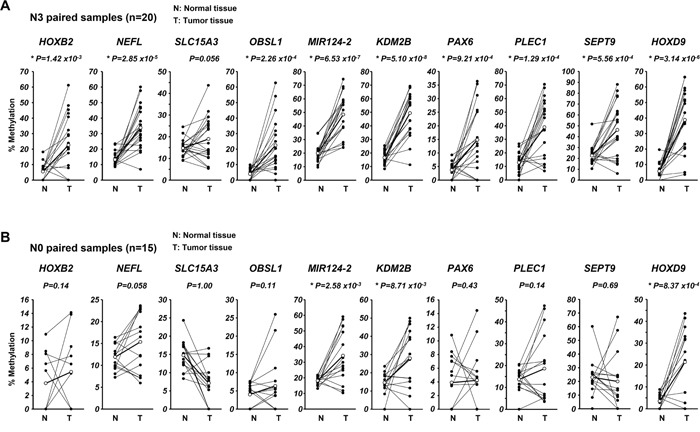
Analysis of DNA methylation of each candidate gene in the 67 ESCC patients of the discovery cohort Differences in methylation status of each candidate gene between paired normal and tumor tissues in N3 samples **(A)** and N0 samples **(B)**. Paired *t*-test was used for comparison of pyrosequencing results for each candidate.

Next, differences in methylation status between non-tumor and tumor tissues were investigated in N0 and N3 samples, and data obtained by pyrosequencing were analyzed by paired t-test. 9 of the 10 candidate genes demonstrated significant differences in methylation status between tumor and non-tumor tissues in the N3 samples (Figure 3A), whereas 3 of the 10 genes showed significant differences in the N0 samples (Figure 3B). Thus, these results suggest those genes may be potentially useful as biomarkers of LNM in ESCC. Moreover, in all N stages, 9 of the 10 genes, except *SLC15A3*, showed significant differences in methylation status between tumor and non-tumor tissues (Supplementary Figure 2). These data suggested that nearly all of the extracted candidate genes may be used as possible predictors of not only LNM, but also the presence of cancer.

The associations between clinicopathological characteristics and methylation status of the candidate genes were analyzed by Pearson's chi-square test. In 9 of the 10 genes, methylation status showed strong correlation with LNM status (Table 1). To evaluate whether methylation status of each candidate gene could predict the presence of LNM, univariate classification analysis was performed, and 8 of the 10 candidate genes showed to have certain level of predictive power. Moreover, ROC analysis and AUC showed moderate accuracy in 7 of the 10 candidate genes (Supplementary Table 4). To identify more powerful candidates for LNM prediction, multivariate analysis was performed using logistic regression and stepwise selection (Table 2). We employed AIC (Akaike's Information Criterion) on the selection in JMP9 software. Conclusively, three candidate genes, *HOXB2*, *SLC15A3* and *SEPT9*, were extracted.

**Table 1:** Correlation between clinicopathological characteristics and methylation status of selected genes in primary ESCC cases

		N	*HOXB2*			*NEFL*			*SLC15A3*			*OBSL1*			*miR-124-2*		
			*cutoff =16.3*			*cutoff =21.3*			*cutoff =13*			*cutoff =12.6*			*cutoff =39*		
			Low	High	*p*-value	Low	High	*p*-value	Low	High	*p*-value	Low	High	*p*-value	Low	High	*p*-value
Total number		67	42	24	* total 66	43	24		32	35		33	34		25	42	
Age																	
Average 65.6	<65	28	18	10	*1.00*	17	11	*0.80*	13	15	*1.00*	15	13	*0.62*	10	18	*1.00*
(46-83)	≧65	39	24	14		26	13		19	20		18	21		15	24	
Gender																	
	Male	62	38	23	*0.65*	39	23	*0.65*	28	34	*0.18*	31	31	*1.00*	23	39	*1.00*
	Female	5	4	1		4	1		4	1		2	3		2	3	
Histological grading																	
	GoodModerate	53	32	20	*0.56*	35	18	*0.55*	25	28	*1.00*	26	27	*1.00*	18	35	*0.35*
	Poor	14	10	4		8	6		7	7		7	7		7	7	
TNM classification																	
pT category																	
	T1+2	7	3	4	*0.25*	4	3	*0.69*	4	3	*0.70*	5	2	*0.26*	0	7	***0.0401***
	T3+4	60	39	20		39	21		28	32		28	32		25	35	
pN category																	
	N-	15	14	0	***0.0011***	14	1	***0.0069***	12	3	***0.0073***	12	3	***0.0087***	9	6	*0.067*
	N+	52	28	24		29	23		20	32		21	31		16	36	
pM category																	
	M0	50	32	17	*0.77*	34	16	*0.38*	23	27	*0.78*	28	22	*0.09*	22	28	*0.08*
	M1	17	10	7		9	8		9	8		5	12		3	14	
pStage																	
	I+II	15	12	2	*0.07*	14	1	***0.0069***	11	4	***0.0388***	12	3	***0.0087***	7	8	*0.55*
	III+IV	52	30	22		29	23		21	31		21	31		18	34	

**Table:** 

		n	*KDM2B*			*PAX6*			*PLEC*			*SEPT9*			*HOXD9*		
			*cutoff =31.8*			*cutoff =16.4*			*cutoff =4.8*			*cutoff =29.8*			*cutoff =41.4*		
			Low	High	*p*-value	Low	High	*p*-value	Low	High	*p*-value	Low	High	*p*-value	Low	High	*p*-value
Total number		67	21	46		40	27		10	57		31	36		47	20	
Age																	
Average 65.6	<65	28	10	18	*0.57*	16	12	*0.80*	5	23	*0.73*	13	15	*1.00*	23	5	*0.10*
(46-83)	≧65	39	11	28		24	15		5	34		18	21		24	15	
Gender																	
	Male	62	19	43	*0.65*	38	24	*0.39*	9	53	*0.57*	29	33	*1.00*	43	19	*1.00*
	Female	5	2	3		2	3		1	4		2	3		4	1	
Histological grading																	
	GoodModerate	53	17	36	*1.00*	32	21	*1.00*	9	44	*0.67*	24	29	*0.77*	37	16	*1.00*
	Poor	14	4	10		8	6		1	13		7	7		10	4	
TNM classification																	
pT category																	
	T1+2	7	3	4	*0.67*	4	3	*1.00*	1	6	*1.00*	3	4	*1.00*	4	3	*0.42*
	T3+4	60	18	42		36	24		9	51		28	32		43	17	
pN category																	
	N-	15	9	6	***0.0110***	13	2	***0.0181***	6	9	***0.0061***	12	3	***0.0037***	15	0	***0.0031***
	N+	52	12	40		27	25		4	48		19	33		32	20	
pM category																	
	M0	50	17	33	*0.55*	32	18	*0.26*	9	41	*0.43*	27	23	***0.0475***	36	14	*0.56*
	M1	17	4	13		8	9		1	16		11	6		11	6	
pStage																	
	I+II	15	8	7	*0.06*	12	3	*0.08*	7	8	***0.0006***	10	5	*0.09*	14	1	***0.0281***
	III+IV	52	13	39		28	24		3	49		21	31		33	19	

p-values are from χ^2^ or Fisher's exact test as appropriate, and were statistically significant when < 0.05 (two-sided).

**Table 2:** Logistic regression analysis and stepwise selection of candidate genes

	Parameter	Intercept	df	Wald	Significance probability
	Intercept	-1.506	1.000	0.000	1.000
*	*HOXB2*	0.097	1.000	2.548	0.110
	*NEFL*	0.000	1.000	1.754	0.185
*	*SLC15A3*	0.085	1.000	2.450	0.118
	*OBSL1*	0.000	1.000	0.189	0.664
	*MIR124-2*	0.000	1.000	0.075	0.785
	*KDM2B*	0.000	1.000	0.107	0.744
	*PAX6*	0.000	1.000	0.085	0.771
	*PLEC*	0.000	1.000	0.021	0.883
*	*SEPT9*	0.028	1.000	1.497	0.221
	*HOXD9*	0.000	1.000	0.765	0.382

*selected candidate gene by logistic regression analysis (stepwise method) df: degree of freedom.

### Methylation status of *HOXB2* and *SEPT9* may be useful as predictive biomarkers for the presence of LNM in ESCC

**Figure 4:**
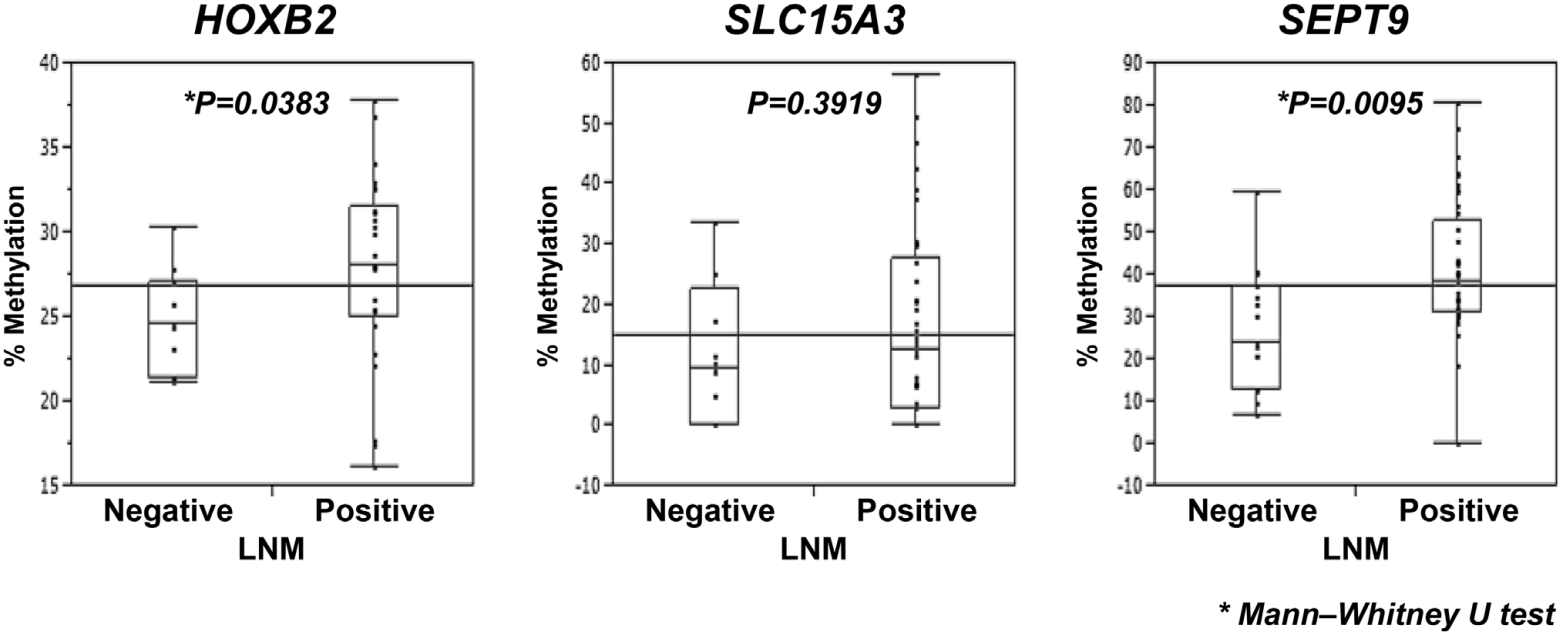
Analysis of DNA methylation of *HOXB2, SLC15A3*, and *SEPT9* in the 59 ESCC patients of the independent set of ESCC cases Validation of the methylation status of three candidate genes, *HOXB2, SLC15A3*, and *SEPT9*, by pyrosequencing in the independent set of ESCC cases. The methylation status in the LNM-negative and -positive groups were analyzed using the Mann–Whitney *U*-test. The horizontal lines represent the means of the whole samples for each genes.

To determine whether *HOXB2*, *SLC15A3*, and *SEPT9* can predict the presence of LNM in another cohort, pyrosequencing analysis of these genes was performed in an independent set of ESCC cases comprising 59 ESCC samples (hereafter called the validation cohort). The clinicopathological characteristics of the validation cohort are shown in Supplementary Table 5. The methylation statuses of *HOXB2* and *SEPT9* showed significant differences between LNM-negative and -positive samples (Figure 4), whereas *SLC15A3* did not. Thus, *HOXB2* and *SEPT9* were identified as possible DNA methylation predictive biomarkers of LNM in ESCC.

## DISCUSSION

We performed a genome-wide screening of DNA methylation status in a discovery cohort of 67 primary ESCC tissues and their paired normal esophageal tissues. We extracted three genes, *HOXB2*, *SLC15A3*, and *SEPT9*, as more powerful candidates predicting LNM in ESCC, using several statistical analyses and pyrosequencing analysis in a discovery cohort. Finally, we confirmed that *HOXB2* and *SEPT9* were highly methylated in LNM-positive tumors in 59 ESCC validation samples. Generally, endoscopic resection, which is a less invasive treatment, is indicated for treatment of early stage ESCC. This indication is based on evidence of reduced LNM possibility within the mucosa of tumor depth (T1a in TNM 7th), as well as the belief that cervical lymph node resection can be omitted in cases of lower esophagus ESCC without obvious LNM. Therefore, accurate predictive biomarkers for LNM are important for the indication of less invasive therapy for ESCC patients. Thus, the present results may be useful for the prediction of LNM in ESCC.

DNA methylation is one of the most heavily studied phenomena of epigenetics, and current research focuses on its usefulness as a clinical diagnostic or prognostic marker, as well as a possible therapeutic target [21]. Our methylation-array analysis identified 10 genes (*HOXB2*, *NEFL*, *SLC15A3*, *OBSL1*, *MIR124-2*, *KDM2B*, *PAX6*, *PLEC*, *SEPT9*, *HOXD9*) as candidates for predictive biomarkers of LNM in a discovery cohort of 67 ESCC cases. In previous studies, 8 of the 10 candidate genes, *HOXB2* [22], *NEFL* [23], *SLC15A3* [24], *MIR124-2* [25], *KDM2B* [26], *PAX6* [27], *SEPT9* [28, 29] and *HOXD9* [30], have been reported to be associated with DNA methylation. To extract candidate genes suitable for clinical use, we performed pyrosequencing analysis, a common method for examining methylation status, and performed univariate and multivariate analyses. Comparing LNM-positive and -negative groups, 9 of the 10 candidate genes showed significant hypermethylation in LNM-positive cases (Table 1). In addition, 3 of the 10 candidate genes, *HOXB2*, *SLC15A3*, and *SEPT9*, were identified as possible predictive biomarkers by multivariate logistic regression analysis with stepwise variable selection (Table 2, Supplementary Table 6). Subsequently, by evaluation of an independent set of ESCC cases (the validation cohort), we concluded that the methylation status of *HOXB2* and *SEPT9* may be able to predict the presence of LNM. Although, we have calculated cut-off value according to their clinical information in each candidate gene (Table 1), we consider them inappropriate to adapt to the analysis for the validation cohort. Because we obtained samples from frozen resected specimen in the discovery cohort and from paraffin embedded specimen in the validation cohort. Since the quality of extracted DNA was different between the discovery cohort and the validation cohort, the cut-off values lead from discovery cohort could not apply to the validation cohort. Finally, we have compared the methylation status of candidates in the validation cohort. We also consider it will be necessary to evaluate the prediction power of combination of these three genes using another independent set of ESCC cases.

We next analyzed methylation status of these candidate genes in the TCGA database. There were 93 ESCC samples without chemotherapy in the database and patients of those included multiracial populations. The result of the statistical analyses showed no significance among three candidates in the dataset (Supplementary Figure 3). We concluded thtat further analyses for the dataset with same race and admirable sample conditions are required.

*HOXB2* encodes a nuclear protein with a homeobox DNA-binding domain [31]. The relationship between *HOXB2* expression and cancer progression has been reported in several studies, and *HOXB2* has been shown to have bifunctional roles, as an oncogene and as a tumor suppressor gene. Overexpression of *HOXB2* was shown to be associated with cancer progression in cervical cancer, pancreatic cancer, and lung adenocarcinoma [32–34]. On the other hand, suppression of *HOXB2* increased tumor growth in mice xenograft models of breast cancer cell lines, and overexpression of *HOXB2* induced apoptotic cell death *in vitro* in acute myeloid leukemia (AML) [35]. In addition, it was reported that expression of *HOXB* cluster genes was repressed by DNA methylation in oral cancer cell lines [22]. At present, there have been no reports on the association between epigenetic regulation of *HOXB2* and LNM in ESCC. In the present study, we examined only methylation status, and not gene expression. Our HM450 methylation array results (Supplementary Figure 1), demonstrated that the methylation status of some probes in tumor tissues were significantly hypermethylated compared with the paired normal tissue, and the intensity of methylation became higher with increasing LNM. Furthermore, pyrosequencing analysis of the validation cohort demonstrated that the methylation status of *HOXB2* was significantly higher in ESCC samples with LNM than those without LNM. Thus, DNA methylation status of *HOXB2* may be a predictive biomarker for the detection of ESCC.

*SEPT9*, a member of the septin family, regulates cytokinesis and the cell cycle, and has multiple splicing variants. It has been reported that *SEPT9* is abnormally expressed in ESCC, ovarian, breast, prostate, and colorectal cancers (CRC) and acts as an oncogene or tumor suppressor gene [28, 29, 36–38]. *SEPT9* is known to be frequently methylated in ESCC and CRC [28, 29]. Detection of methylated *SEPT9* in blood plasma has been used as an aid in the diagnostic evaluation of CRC [29]. This method can detect presence of CRC at all stages, whereas almost adenomas were unable to be detected. Our data showed that methylation of *SEPT9* significantly differed between normal and ESCC tumor tissues of all stages (Supplementary Figure 2), and the presence of LNM in ESCC could be predicted by *SEPT9* methylation status with significance (Figure 3 and 4). Thus, in addition to *HOXB2*, methylation of *SEPT9* may be a predictive biomarker in ESCC.

*SLC15A3* encodes a protein involved in the transport of glucose and other sugars, bile salts, organic acids, metal ions, and amine compounds [39]. However, there are few studies reporting an association between *SLC15A3* and cancer or methylation [24, 40]. In our discovery cohort, methylation status of *SLC15A3* significantly differed between LNM-positive and -negative samples. However, in the validation cohort, although the methylation status of SLC15A3 in LNM-positive samples tended to be higher than that of the LNM-negative samples, this difference was not significant. This may be partially due to sample problems or a batch effect, because the DNA in the validation cohort was extracted from paraffin-embedded samples, whereas the DNA of the discovery cohort was extracted from frozen tissue samples.

In conclusion, the present results suggest that DNA methylation of *HOXB2* and *SEPT9* may be useful as predictive biomarkers of LNM in ESCC. The presence of LNM in ESCC is highly associated with poor prognosis, and prediction of LNM presence before treatment is important in clinical practice. Clinically, because the use of LNM predictive methylation biomarkers requires only a small amount of DNA from the tumor tissue of the ESCC patient, the diagnosis of the presence of LNM should be easy and may be applicable for use with liquid biopsies. Use of such biomarkers may also allow detection of micrometastases unable to be detected by CT. In the present study, we investigated the association between methylation status and presence of LNM in ESCC, and observed that the methylation status of *HOXB2* and *SEPT9* may be useful as diagnostic or prognostic biomarkers in ESCC.

## MATERIALS AND METHODS

### Primary tumor samples

A total of 67 ESCC frozen primary tumors and paired non-cancerous tissue samples were obtained from patients with ESCC had undergone esophagectomy with lymph node dissection at Tokyo Medical and Dental University between 2005 and 2010 (discovery cohort) (Supplementary Table 1). After approval by the local ethics committee of the Medical Research Institute and Faculty of Medicine, Tokyo Medical and Dental University, formal written consent was obtained from all patients. The average age of the discovery cohort was 65.6 years [range, 46-83 years], and comprised 62 males and 5 females. No patients had undergone prior chemotherapy or radiotherapy. Lymph node metastasis status was N0 in 15 patients, N1 in 15 patients, N2 in 17 patients, and N3 in 20 patients. Additionally, 59 paraffin-embedded ESCC tumor samples from patients who had undergone esophagectomy at Kyoto Prefectural University of Medicine between 2003 and 2013 were included as an independent set of ESCC cases for the validation. After approval by the local ethics committee of Kyoto Prefectural University of Medicine, formal written consent was obtained from all patients. In this cohort, T1 (TNM classification) cases were omitted due to the difficulty of extracting DNA from the paraffin-embedded samples. Specimens were classified by the TNM classification of the Union for International Cancer Control (UICC 7th) [41].

### DNA extraction and bisulfite treatment

DNA from frozen samples were extracted by the phenol chloroform method and treated by bisulfite conversion with the EZ DNA Methylation kit (Zymo Research) according to the manufacturer's recommendations. DNA from paraffin-embedded samples were extracted using the DNeasy Blood & Tissue kit (QIAGEN, KJ Venlo, Netherlands) according to the manufacturer's recommendations.

### Illumina infinium HumanMethylation450 BeadChip array

For the discovery round, the Human Methylation450 BeadChip (Illumina, San Diego, CA, USA), which covers 96% of the known CpG islands and 485,577 CpG sites, was employed. Approximately 500 ng of bisulfite-converted DNA from the discovery cohort was applied to the array and analyzed. The GenomeStudio software (Illumina) was used for the quality check of the raw data of each probe and for data normalization. We did not remove SNP-associated probes and those corresponding to the sex chromosomes. Extracted data were analyzed by JMP9 (SAS Institute Inc., Cary, NC, USA).

### Pyrosequencing for quantitative measurement of DNA methylation level and statistical analysis

The DNA methylation statuses of the 10 candidate genes that were extracted from the discovery cohort previously analyzed by Illumina Human Methylation450 were validated by pyrosequencing analysis. Primers for pyrosequencing assays were designed by PyroMark Assay Design software (version 2.0.1.15 QIAGEN). PCR was performed under standard conditions with biotin-labeled primers using the PyroMark PCR kit and following the manufacturer's instructions. The biotin-labeled PCR products were assessed by electrophoresis and subjected to the PyroMark Vacuum Prep Tool (Biotage, Sweden) and PyroMark Q96 ID pyrosequencer (QIAGEN). The pyrosequencing data were analyzed by PyroMark Q96 software (version 2.5.8) and JMP9. Uni- and multivariate analyses and logistic regression analysis were performed using JMP9. Multiplicity corrections were performed by BH method.

## SUPPLEMENTARY MATERIALS FIGURES AND TABLES


