# Supplementary material for: Genome-wide screening of DNA methylation associated with lymph node metastasis in esophageal squamous cell carcinoma
Source: Oncotarget. 2017 Apr 17;8(23):37740–50. doi: 10.18632/oncotarget.17147 (PMC5514945; doi:10.18632/oncotarget.17147)
Supplement: Supplementary file 1 [file oncotarget-08-37740-s001.pdf]

Genome-wide screening of DNA methylation associated with lymph node metastasis in esophageal squamous cell carcinoma

SUPPLEMENTARY FIGURES AND TABLES

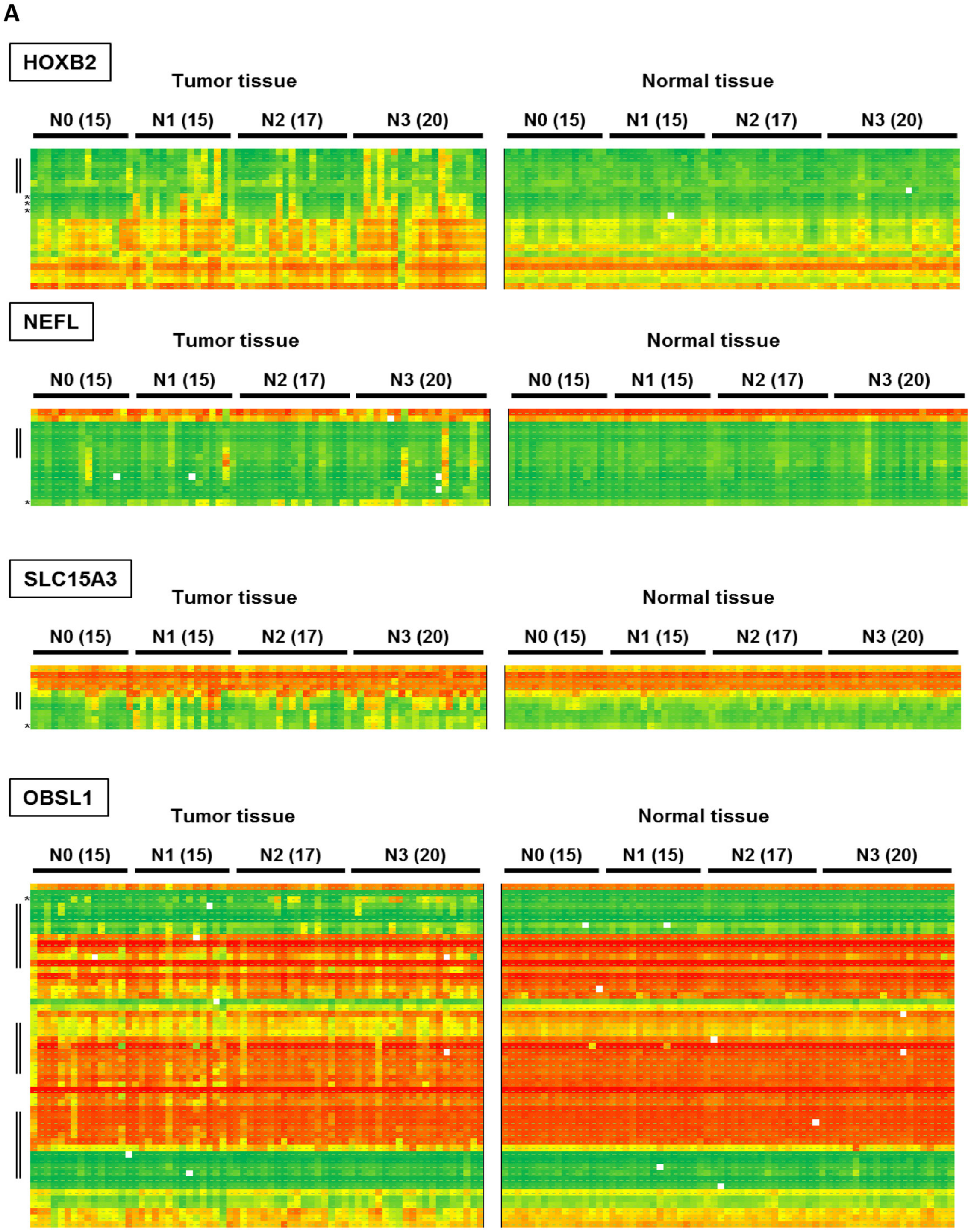

(*Continued*)

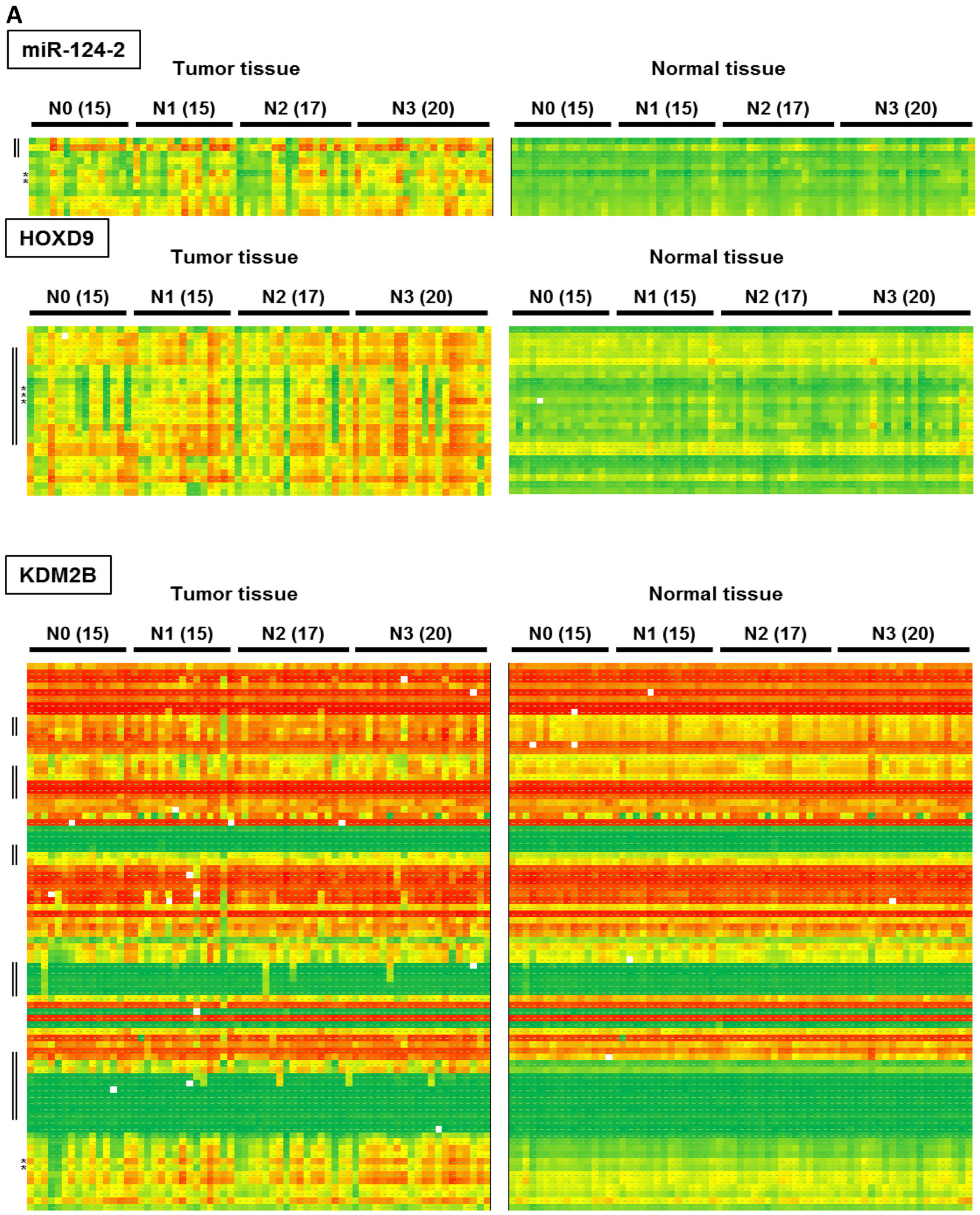

A
miR-124-2
Tumor tissue
Normal tissue
N0 (15)
N1 (15)
N2 (17)
N3 (20)
HOXD9
Tumor tissue
Normal tissue
N0 (15)
N1 (15)
N2 (17)
N3 (20)
KDM2B
Tumor tissue
Normal tissue
N0 (15)
N1 (15)
N2 (17)
N3 (20)

(Continued)

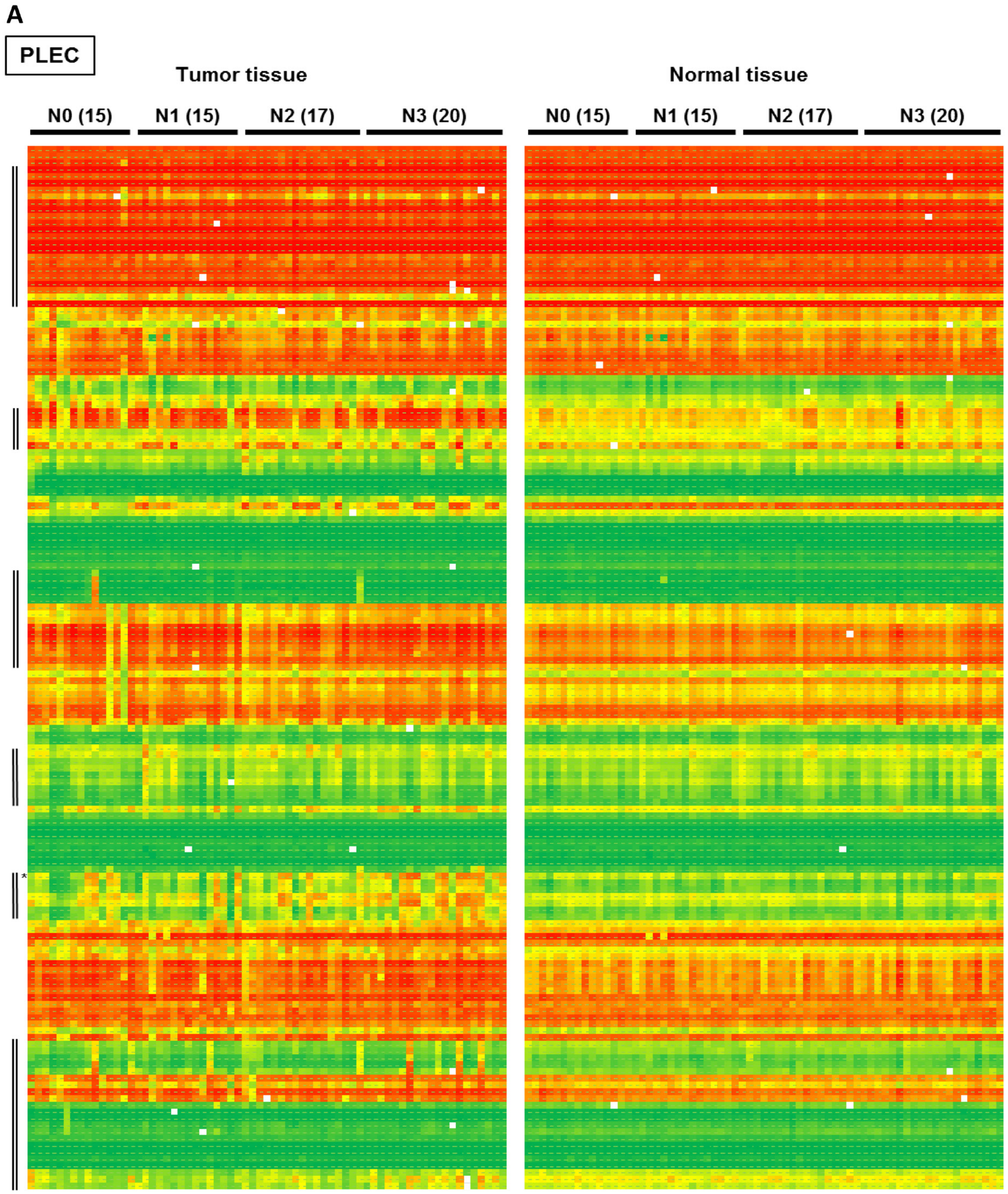
A
PLEC
Tumor tissue
Normal tissue
N0 (15)
N1 (15)
N2 (17)
N3 (20)
N0 (15)
N1 (15)
N2 (17)
N3 (20)
*

(*Continued*)

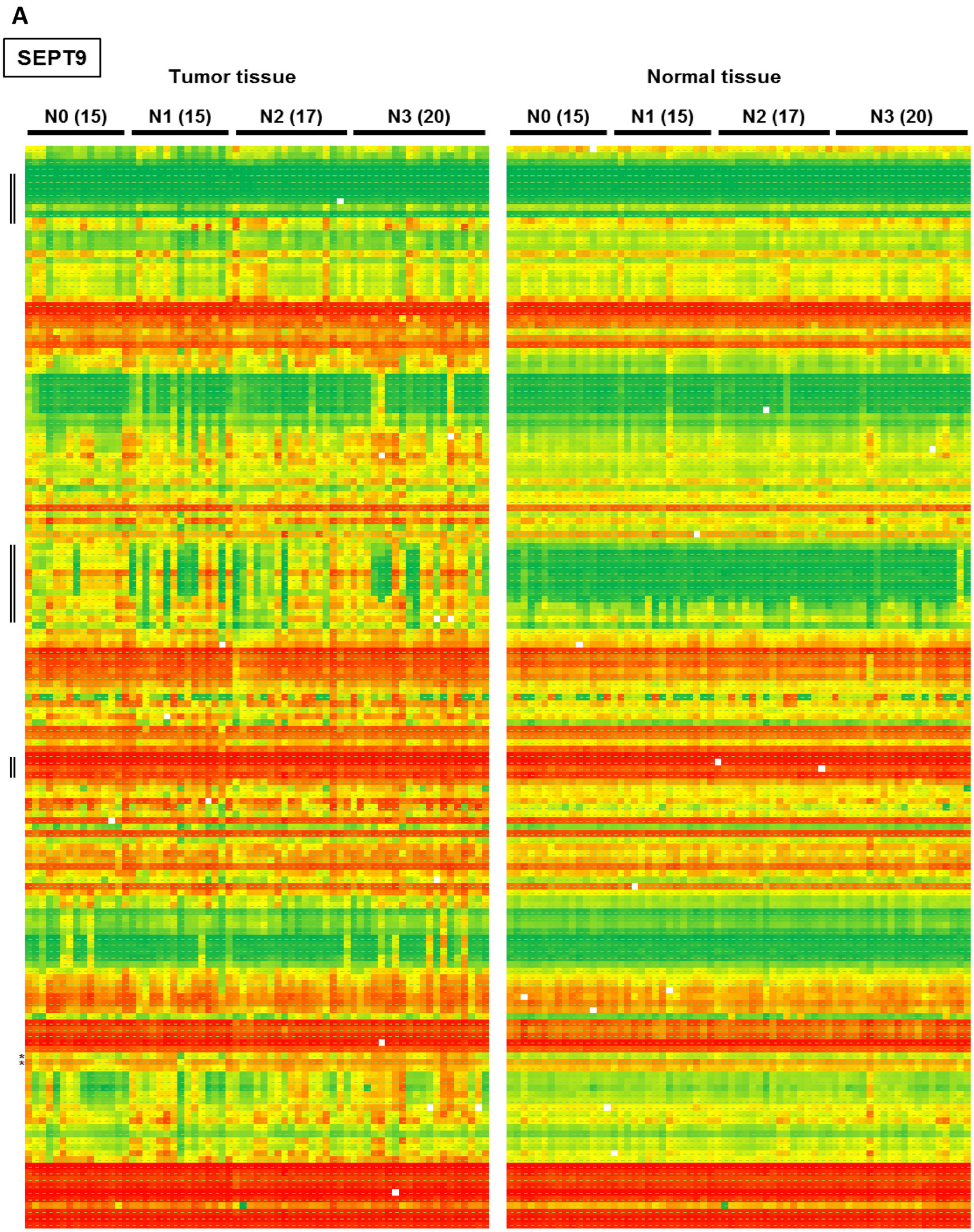
A
SEPT9
Tumor tissue
Normal tissue
N0 (15)
N1 (15)
N2 (17)
N3 (20)
N0 (15)
N1 (15)
N2 (17)
N3 (20)

(*Continued*)

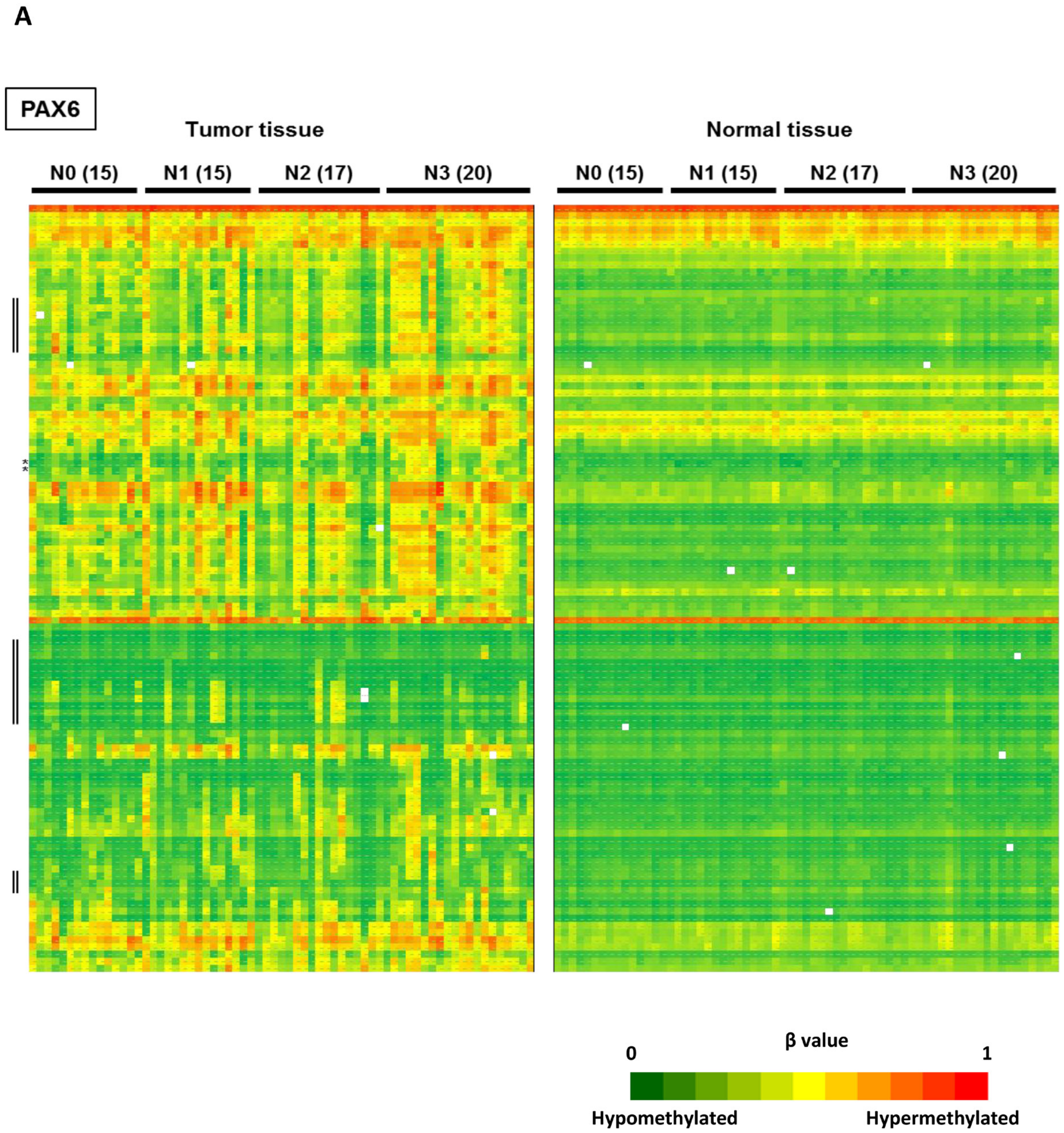
A
PAX6
Tumor tissue
Normal tissue
N0 (15)
N1 (15)
N2 (17)
N3 (20)
N0 (15)
N1 (15)
N2 (17)
N3 (20)
β value
0
1
Hypomethylated
Hypermethylated

(*Continued*)

B

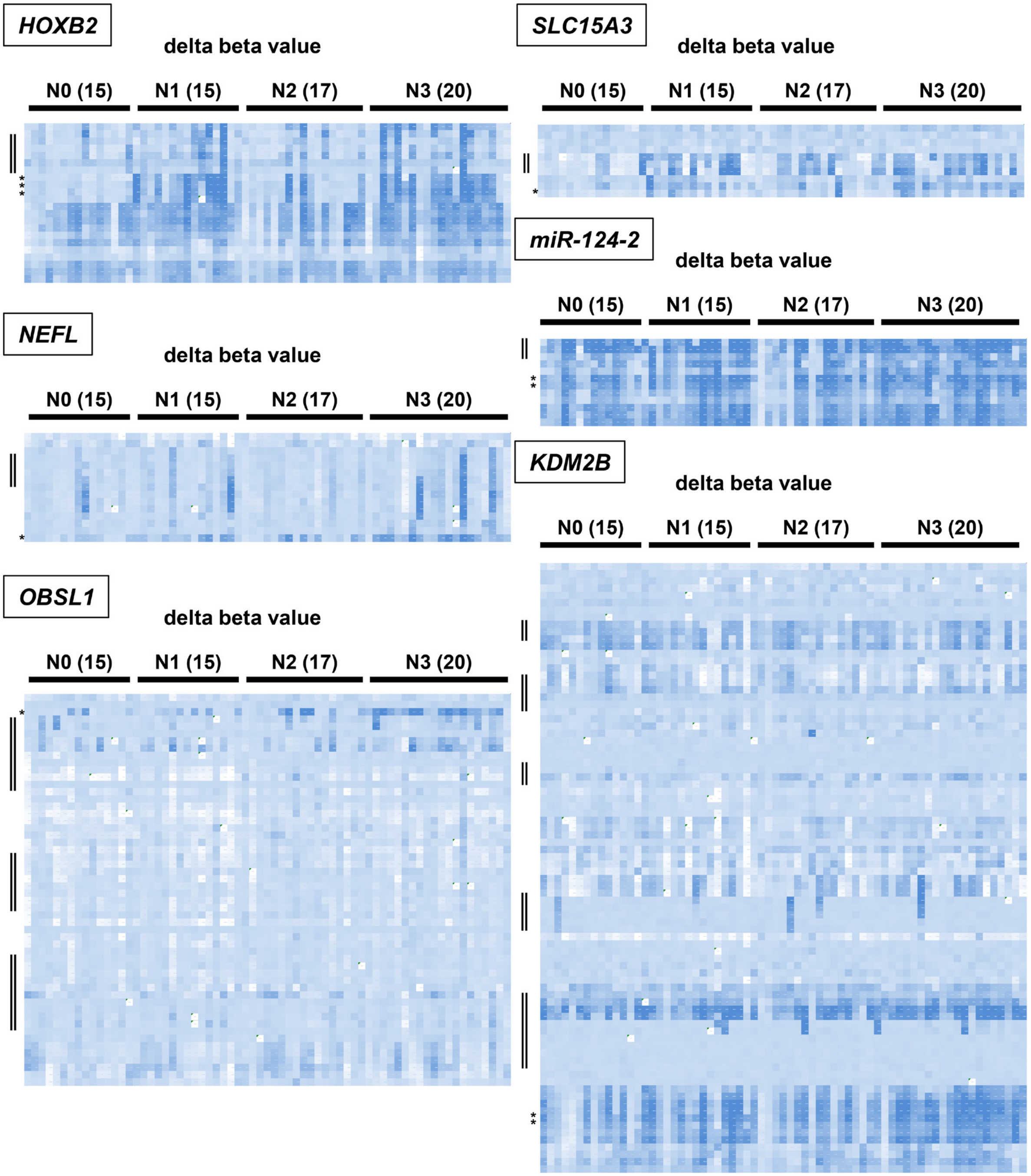
HOXB2
delta beta value
N0 (15)
N1 (15)
N2 (17)
N3 (20)
SLC15A3
delta beta value
N0 (15)
N1 (15)
N2 (17)
N3 (20)
miR-124-2
delta beta value
N0 (15)
N1 (15)
N2 (17)
N3 (20)
NEFL
delta beta value
N0 (15)
N1 (15)
N2 (17)
N3 (20)
KDM2B
delta beta value
N0 (15)
N1 (15)
N2 (17)
N3 (20)
OBSL1
delta beta value
N0 (15)
N1 (15)
N2 (17)
N3 (20)

(Continued)

B

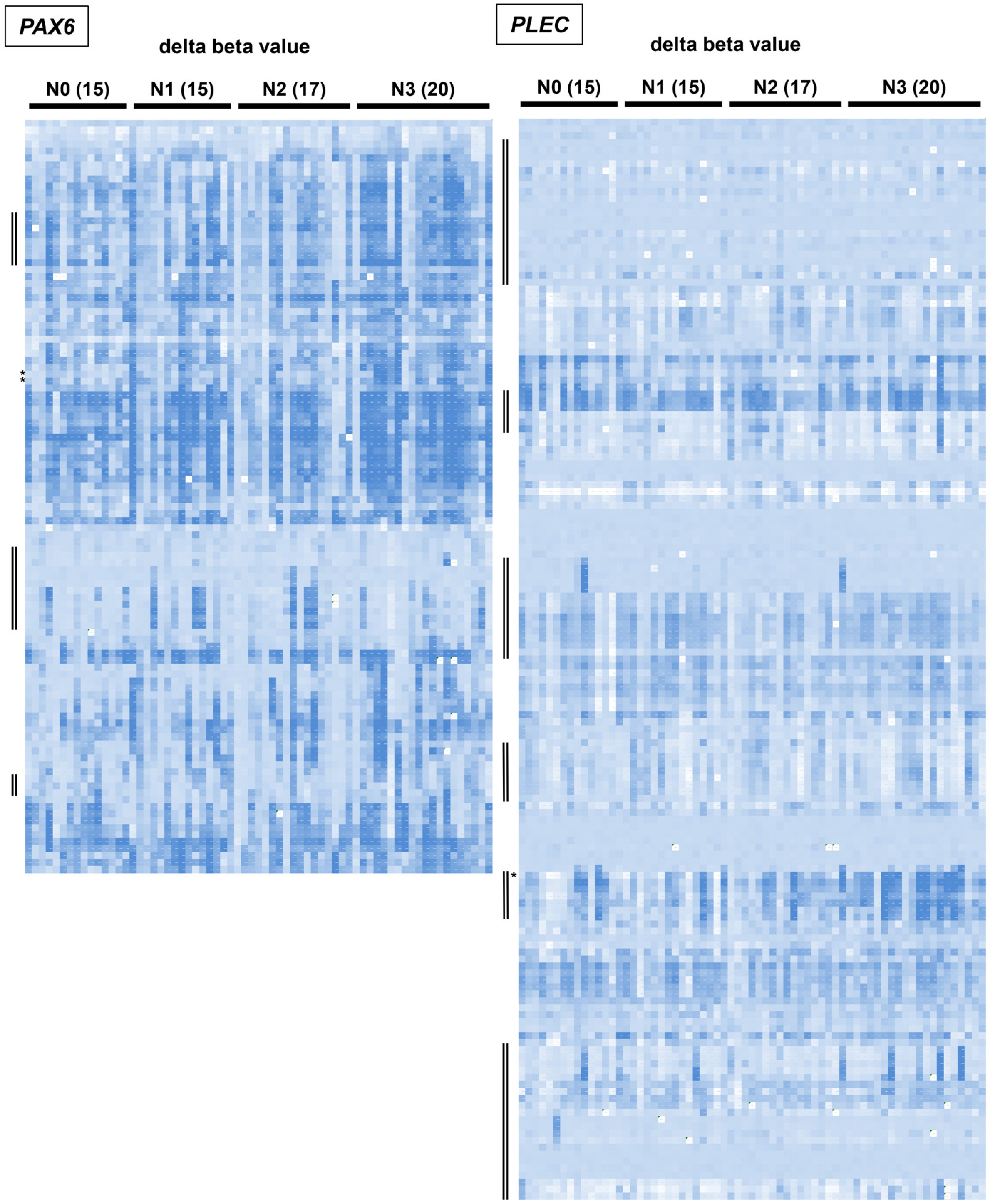
PAX6
delta beta value
N0 (15)
N1 (15)
N2 (17)
N3 (20)
PLEC
delta beta value
N0 (15)
N1 (15)
N2 (17)
N3 (20)

(*Continued*)

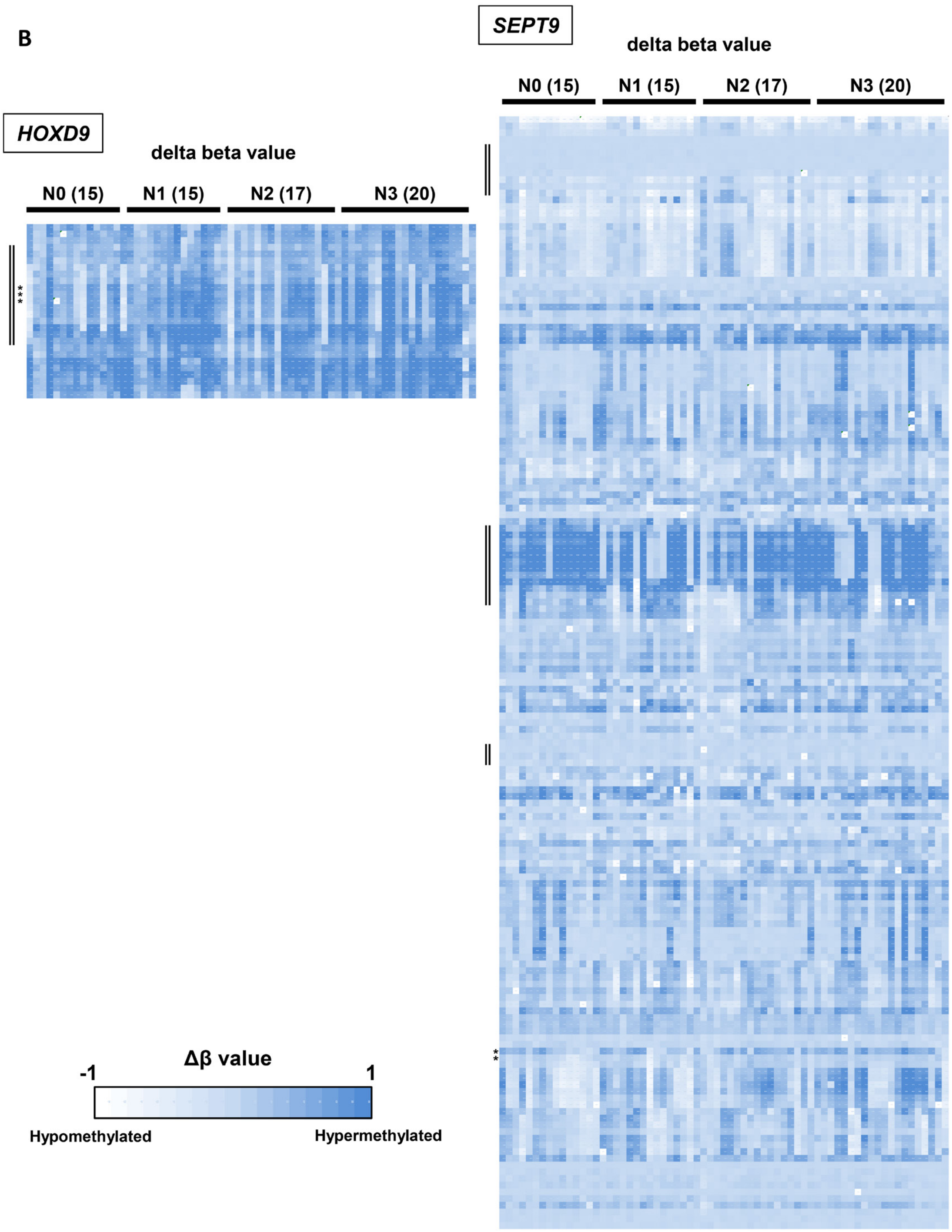

Supplementary Figure 1: Screening epigenetic biomarkers for prediction of the presence of LNM in primary ESCC cases using the Illumina Infinium HumanMethylation450 BeadChip. **(A)** Raw data from DNA methylation analysis of 67 paired ESCC samples using the Illumina HumanMethylation450 BeadChip. One pixel in this matrix represents the beta value, which ranges from 0 to 1. As shown in the lower right panel, red boxes (beta value=1) represent hypermethylation, and green (beta value=0) represents hypomethylation. In each row, samples were ordered by LNM status according to the UICC 7th version. The numbers next to “N stage” indicate the number of samples. In each sequence, the array probes were ordered by the Illumina probe ID. **(B)** The heat map showing the delta beta values calculated from the raw *HOXB2* gene and the formula indicated on the lower middle panel. As shown in the lower right panel, boxes indicate hypermethylation (dark blue; delta beta value=1) and hypomethylation (white; delta beta value=-1).

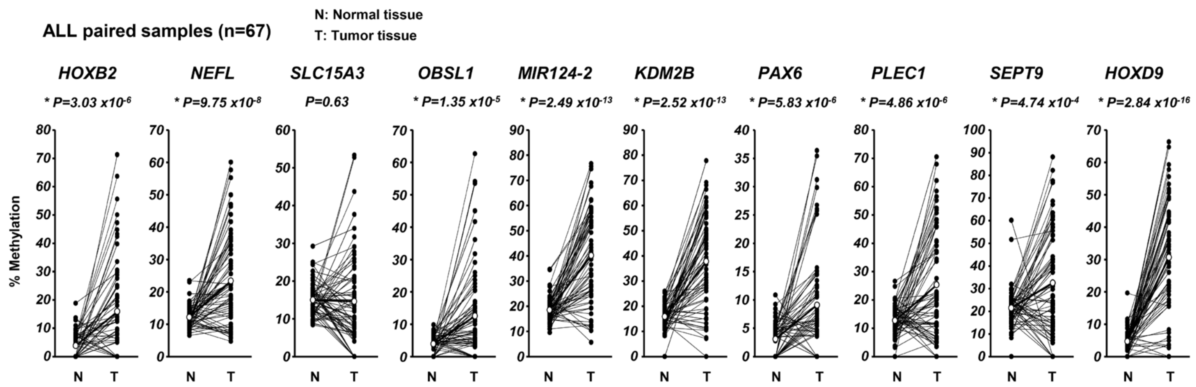

Supplementary Figure 2: Analysis of DNA methylation of each candidate gene in the 67 ESCC patients of the discovery cohort. Comparison of methylation status between paired normal and tumor tissues. Mann-Whitney *U*-test was used for analysis.

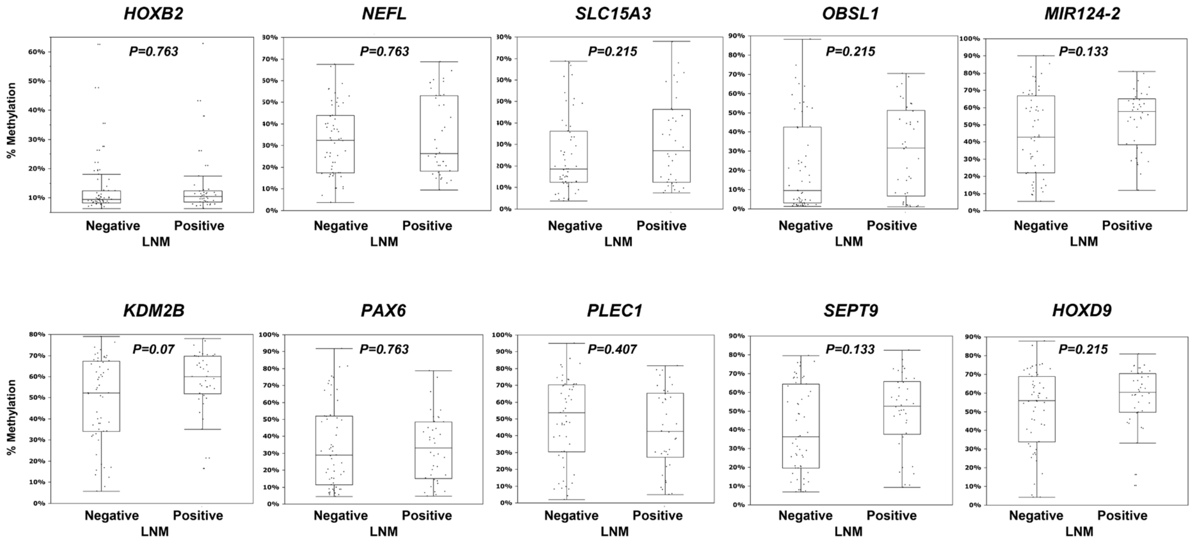

Supplementary Figure 3: Analysis of DNA methylation of each candidate gene in the 93 ESCC patients in TCGA database. Comparison of methylation status between tissues with LNM positive and negative patients with ESCC. The data was downloaded from TCGA database. *Welch t-test* was used for analysis.

Supplementary Table 1: Characteristics of the 67 ESCC patients of the discovery cohort

		N	%
Total number		67	
Age			
(average: 65.6, range: 46-83)	< 65	28	41.8%
	≧65	39	58.2%
Gender			
	Male	62	92.5%
	Female	5	7.5%
Histological grading			
	Good	18	26.9%
	Moderate	35	52.2%
	Poor	14	20.9%
TNM classification			
pT category			
	T1	3	4.5%
	T2	4	6.0%
	T3	47	70.1%
	T4	13	19.4%
pN category			
	N0	15	22.4%
	N1	15	22.4%
	N2	17	25.4%
	N3	20	29.9%
pM category			
	M0	50	74.6%
	M1	17	25.4%
pStage			
	I	1	1.5%
	II	14	20.9%
	III	35	52.2%
	IV	17	25.4%

Supplementary Table 2: LNM prediction marker candidates extracted from Illumina Human Methylation 450 data

Target ID[1]	Gene		Location	Major functions
cg12121660<br>cg17807663<br>cg10788618	HOXB2	homeobox B2	17q21.32	transcription factor activity
cg01432561	NEFL	neurofilament, light polypeptide	8p21.2	structural constituent of cytoskeleton
cg18151345	SLC15A3	solute carrier family 15, member 3	11q12.2	transporter activity
cg14535068	OBSL1	obscurin-like 1	2q35	cytoskeletal adaptor activity
cg25900085<br>cg05474726<br>cg27313642	MIR124-2	microRNA 124-2	8q12.3	not known
cg15234492<br>cg17452615	KDM2B	lysine (K)-specific demethylase 2B	12q24.31	oxidoreductase activity, histone demethylase activity
cg04938549<br>cg27011060	PAX6	paired box 6	11p13	protein kinase binding, histone acetyltransferase binding
cg24507266	PLEC1	plectin	8q24.3	ankyrin binding, structural constituent of muscle
cg19948701<br>cg15229994	SEPT9	septin 9	17q25.2	GTP binding, GTPase activity
cg09968620<br>cg14991487<br>cg09578028	HOXD9	homeobox D9	2q31.1	transcription factor activity

[1] Illumina unique probe identifier.

Supplementary Table 3: Primer and probe sequences for pyrosequencng analysis

Assay		Forward primer (5'-3')		Reverse primer (5'-3')
HOXB2		AGGGAGATTGGGTTTATAAATAGTT	○	AATTTCTTCTCTTTCATCCAAAAAAACTC
NEFL	○	ATGAAATTTGGGGGATTTTTAATATTATG		AAAAACTCCACACCCAAACTTCTTCAA
SLC15A3		AGTGAATATGGGGTTGAGATG	○	ACCCTACCCCCAACTCCTAAATAACA
OBSL1		GGTGTTGGAGGTGATTGT	○	CCTACCCCTACCCAATTAACTTTACTC
MIR124-2	○	TGTGTTGTAAATGGTATGGAGATAT		CATAACTCATAAACCCAACTCCTATC
KDM2B		AGTGGATAGGGATTTAATGATATTTATG	○	ATTCTACCCCCCTCTATAAACTCCTTTACT
PAX6		GATAAAGGAGGGATGGGGAATATTATGA	○	ATCCAAATTTCCCCACCTCCAATACAA
PLEC		GGATTTTGTTTGGTGGTGGTATA	○	TACACCCAACCAAAAATCACTATATAAT
SEPT9		AGTATTTTGGGAGGTTAAGGT	○	CCCCCCCACACAAAATTTATA
HOXD9		GGGTATATGGATTTGGGTTTG	○	AACTACAACCTCCACCATTAATC
**Assay**		**Sequencing primer probe**		**Sequence to analyze**
HOXB2		AAGAGAGTAGAAGATGGGTTTG		TTTTGTYGTYGTTATYGTYGTYGTTATTTTTYGTTGTTTTTTYGGTTTT
NEFL		CAAAAAACACCCAAATACTT		AAAACTAACRCACRCRATAAC
SLC15A3		GGTTGATTTTGATTTAGTTTG		AGGTYGGTGTTTTTTTTAATTTYGATTTATTTGTGGTTTTTTATGGTTYGTAGGTTT
OBSL1		TGGAGGTGATTGTGT		TTYGTTYGGGGGGTTAYGTGTGTTGGTTGYGGGAGGGGGTYGAGTTGTGTTYGG
MIR124-2		CTACCAAAACCCCTATTAATTAATC		TATTCCRTAAAAATATAAACRATACRTATACCTACRTATACATATACATATATCTCCATAC
KDM2B		TTTTTAGTTTTTGAGTATTTATTTG		TTTTTTTTGTYGAGYGTAAGAATTTTTAAAAAATGTTTAAT
PAX6		GAAATAAAGAGAGTAGTGGTTG		GTGYGAATYGATTTTYGGTTTTAAGTTTTATTTGYGGTGGTYGGA
PLEC		TTTAGTTTAGGAGTAGATATGGAT		TTTTYGYGAGTTATTTAGAAYGAGATTAG
SEPT9		ATTTGTTTATTTATTTATTAGATT		TTTTGTYGGGGTTTGTYGTGAGTTTTAYGTTGTTTTGAAGAAAGTYGGGTT
HOXD9		TTTTAGTTTAGAGGATTGATTTAT		TTTYGGTATTGGTAAATATGATTAYGTGGGTYGYGYGATTAATGGTGGAGGTTGTAGT

○ biotin-labeled.

Supplementary Table 4: Univariate analysis and receiver operating characteristic (ROC) analysis of pyrosequencing data for N-/N+ prediction

Gene	Univariate analysis								ROC analysis					
	LN metastasis +			LN metastasis -			*p-value*	*Adjusted p-value*						
	Mean		SE	Mean		SE			Cut-off	AUC	Sensitivity	Specificity	PPV	NPV
HOXB2	19.43	±	2.43	5.37	±	1.55	**$7.30x10^{-6}$	**$7.30x10^{-5}$	16.31	0.76***	0.46	1.00	1.00	0.33
NEFL	21.07	±	1.79	11.53	±	1.98	**$9.54x10^{-4}$	**$3.18x10^{-3}$	21.26	0.73***	0.44	0.93	0.96	0.33
SLC15A3	18.43	±	1.72	8.72	±	1.50	**$8.61x10^{-5}$	**$4.31x10^{-4}$	12.99	0.74***	0.62	0.80	0.91	0.38
OBSL1	19.19	±	2.13	10.07	±	2.37	**$6.68x10^{-3}$	*0.0134	12.62	0.71***	0.62	0.82	0.91	0.42
MIR124-2	45.03	±	2.21	38.51	±	3.15	0.1024	0.1138	38.99	0.63	0.69	0.60	0.86	0.36
KDM2B	44.26	±	2.49	30.90	±	4.32	*0.0132	*0.0188	31.83	0.71***	0.77	0.60	0.87	0.43
PAX6	17.32	±	1.60	10.95	±	1.32	**$3.40x10^{-3}$	**$8.51x10^{-3}$	16.43	0.65	0.48	0.87	0.93	0.37
PLEC	23.41	±	2.41	16.05	±	4.14	0.1376	0.1376	4.81	0.65	0.92	0.40	0.84	0.54
SEPT9	36.49	±	2.98	20.27	±	5.50	*0.0163	*0.0204	29.76	0.73***	0.65	0.80	0.92	0.40
HOXD9	35.02	±	2.25	22.36	±	3.92	**$9.91x10^{-3}$	*0.0165	41.42	0.71***	0.54	0.80	0.90	0.33

*p< 0.05 **p< 0.01 *Welch t-test* ***AUC ≧ 0.7: Moderate Accuracy.

Supplementary Table 5: Characteristics of the 59 ESCC patients of the independent set of ESCC cases

		N	%
Total number		59	
Age			
(average: 67.1, range: 46-88)	<65	24	40.7%
	≧65	35	59.3%
Gender			
	Male	45	76.3%
	Female	14	23.7%
TNM classification			
pT category			
	T1	0	0.0%
	T2	17	28.8%
	T3	37	62.7%
	T4	5	8.5%
pN category			
	N0	16	27.1%
	N1	13	22.0%
	N2	22	37.3%
	N3	8	13.6%
pM category			
	M0	51	86.4%
	M1	8	13.6%
pStage			
	I	8	13.6%
	II	12	20.3%
	III	32	54.2%
	IV	7	11.9%

Supplementary Table 6: Step history of logistic regression analysis and selection of candidate genes

Step	Parameter	Action	*seqSS	**SigProb	Rsquare	***p	****AICc	*****BIC
1	*HOXB2*	entered	11.911	0.001	0.175	2	60.490	64.679
2	*SLC15A3*	entered	4.062	0.044	0.234	3	58.625	64.807
3	*SEPT9*	entered	2.654	0.103	0.273	4	58.239	66.342
4	*NEFL*	entered	1.467	0.226	0.295	5	59.116	69.065
5	*HOXD9*	entered	0.667	0.414	0.304	6	60.873	72.588
6	*OBSL1*	entered	0.319	0.572	0.309	7	63.061	76.458
7	*PAX6*	entered	0.229	0.632	0.312	8	65.427	80.418
8	*KDM2B*	entered	0.167	0.683	0.315	9	67.948	84.441
9	*MIR124-2*	entered	0.088	0.767	0.316	10	70.646	88.543
10	*PLEC*	entered	0.021	0.883	0.316	11	73.513	92.711
11	best	specific			0.273	4	58.239	66.342

***sequential sum of squares.**
****significance probability.**
*****number of parameters.**
******corrected Akaike's Information Criterion.**
*******Bayesian Information Criterion.**
